# A Case of Premature Ventricular Contraction-Induced Cardiomyopathy

**DOI:** 10.7759/cureus.91034

**Published:** 2025-08-26

**Authors:** Michael Mazar, Shipra Hingorany

**Affiliations:** 1 Cardiology, University of California, Los Angeles, David Geffen School of Medicine, Los Angeles, USA

**Keywords:** dilated cardiomyopathy, non-ischemic cardiomyopathy, premature ventricular contraction (pvc), pvc ablation, pvc cardiomyopathy

## Abstract

Premature ventricular contractions (PVCs) are often felt to be benign, but can lead to PVC-induced cardiomyopathy when they occur frequently. This case illustrates the progression from asymptomatic PVCs to dilated cardiomyopathy and subsequent recovery following catheter ablation. A 68-year-old male presented with PVCs identified on a routine electrocardiogram. Initial evaluation revealed a PVC burden of 6%, normal left ventricular ejection fraction (LVEF), and no structural heart disease. Over the subsequent 18 months, he remained asymptomatic but developed a 27% PVC burden, global hypokinesis, and a reduced LVEF of 30-35%. Extensive testing (including cardiac catheterization, cardiac MRI, and genetic testing) ruled out ischemic, infiltrative, and genetic causes. Guideline-directed medical therapy (including a beta blocker, angiotensin receptor blocker, and a mineralocorticoid receptor antagonist) was initiated, and the patient abstained from alcohol. Due to persistently high PVC burden and impaired LVEF, he underwent catheter ablation of PVCs originating from the left ventricular summit. Three months later, his PVC burden on 14-day event monitoring fell to <1% and LVEF improved to 40-45%. Over the following two years, the patient underwent additional atrial fibrillation ablation and optimized medical therapy. His LVEF eventually normalized to 55-60% and PVCs remained suppressed. This case illustrates the clinical course and reversibility of PVC-induced cardiomyopathy with appropriate lifestyle changes, medical therapy, and catheter ablation. Early identification and management of frequent PVCs are essential in preventing progression to cardiomyopathy.

## Introduction

Premature ventricular contractions (PVCs) are a common finding on electrocardiograms and are often considered benign, particularly in individuals without structural heart disease. However, in certain cases, frequent PVCs (i.e., greater than 10% PVC burden) can lead to a reversible form of cardiomyopathy known as PVC-induced cardiomyopathy [1]. The development of PVC-induced cardiomyopathy is associated with a high PVC burden resulting in significant left ventricular dysfunction if left untreated [2]. Early recognition and appropriate intervention, including medical therapy, lifestyle modification, and catheter ablation, can restore normal cardiac function. Of note, PVC-induced cardiomyopathy is a diagnosis of exclusion and can only be made after other causes have been ruled out (including ischemic and infiltrative cardiomyopathies). This case highlights the progression of initially asymptomatic PVCs to dilated cardiomyopathy in an otherwise healthy individual and demonstrates the potential for complete recovery of left ventricular function following targeted therapy, underscoring the importance of vigilance in the evaluation and management of patients with frequent PVCs.

## Case presentation

**Figure 1:**
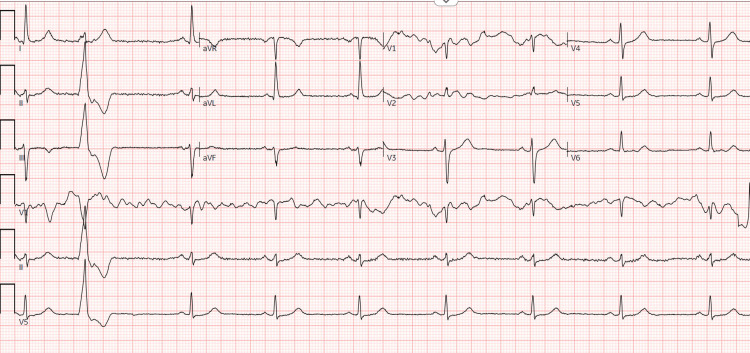
ECG on presentation.

A 68-year-old male was referred to cardiology for the finding of PVCs. He was initially evaluated by his primary care physician, who performed a routine electrocardiogram (ECG). The ECG demonstrated sinus bradycardia at 52 beats per minute (BPM) with occasional PVCs and borderline voltage criteria for left ventricular hypertrophy (Figure 1).

The patient denied any symptoms of chest pain, shortness of breath, or syncope. His past medical history was significant for anxiety, and he reported consuming two to four alcoholic drinks a day.

Further evaluation with an echocardiogram revealed normal left ventricular size, preserved left ventricular systolic function, no valvular pathology, normal wall thickness, and a normal estimated pulmonary artery systolic pressure. A stress echocardiogram showed no evidence of ischemia but was notable for frequent PVCs, including couplets, bigeminy, and trigeminy. The patient denied anginal symptoms after achieving 10.1 metabolic equivalents (METs) of exercise on a treadmill using a standard Bruce protocol. A 14-day heart monitor demonstrated sinus rhythm with a 6% PVC burden. Laboratory findings were within normal limits, including a complete blood count, comprehensive metabolic panel, magnesium, hemoglobin A1c, and thyroid-stimulating hormone levels. His low-density lipoprotein (LDL) cholesterol was mildly elevated at 113 mg/dL. As the patient remained asymptomatic, he was advised to reduce his alcohol intake, which he successfully decreased to 0-2 alcoholic drinks per day.

**Figure 2:**
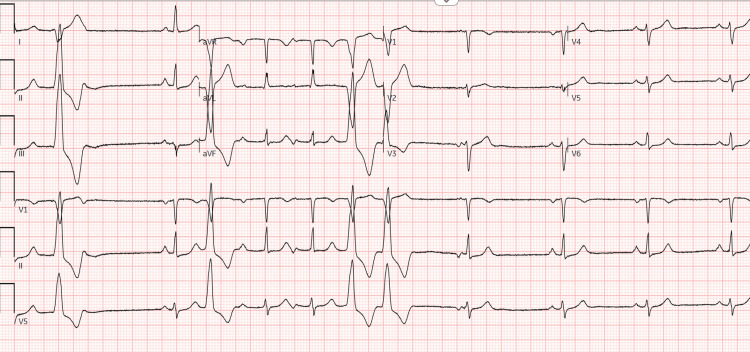
Subsequent ECG demonstrating frequent premature ventricular contractions.

A year and a half later, during a routine clinic visit, the patient remained asymptomatic but was found to have frequent PVCs on a 12-lead ECG (Figure 2).

A repeat 14-day heart monitor revealed sinus rhythm with a significantly increased PVC burden of 27%. He was started on metoprolol succinate at 25 mg once daily. Repeat echocardiography demonstrated a decline in his left ventricular systolic function with global hypokinesis and a left ventricular ejection fraction (LVEF) of 30%-35%. At this point, he eliminated all alcohol consumption, and a cardiac catheterization was arranged to exclude an ischemic cardiomyopathy. His left heart catheterization demonstrated normal coronary arteries and normal left ventricular filling pressures. A cardiac PET scan and cardiac MRI were then completed and were negative for sarcoidosis or other infiltrative diseases.

**Figure 3:**
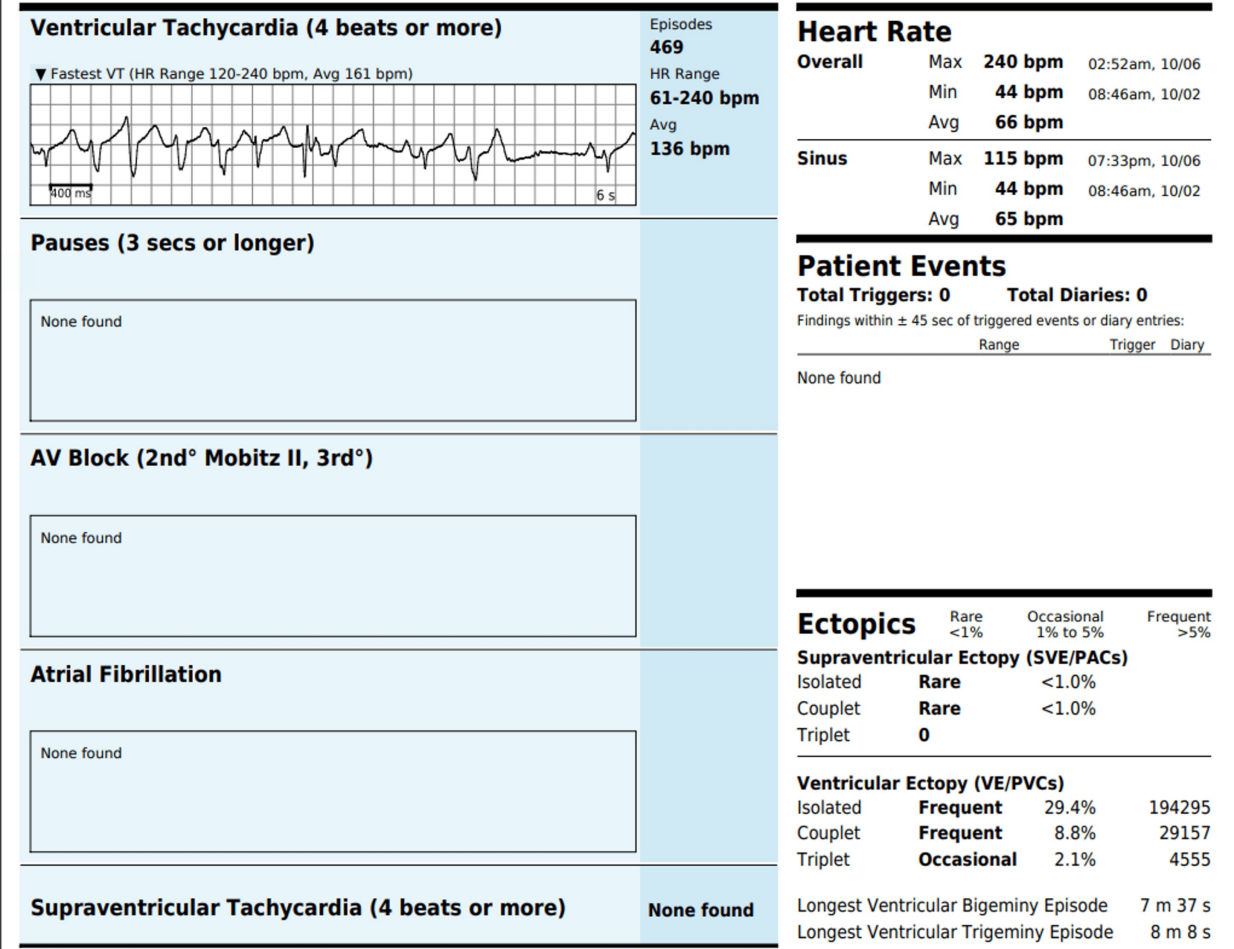
Event monitor demonstrating 29% PVC burden. PVC: premature ventricular contraction; VT: ventricular tachycardia; HR: heart rate; AV: atrioventricular; PACs: premature atrial contractions.

His metoprolol succinate was increased to 50 mg twice daily, and losartan 25 mg daily and spironolactone 25 mg daily were initiated. However, comprehensive guideline-directed medical therapy was limited due to persistently low systolic blood pressure readings below 100 mmHg. Three months later, a repeat echocardiogram showed no improvement, with the LVEF remaining at 30%-35%. A repeat 14-day heart monitor demonstrated a PVC burden of 29% (Figure 3).

**Figure 4:**
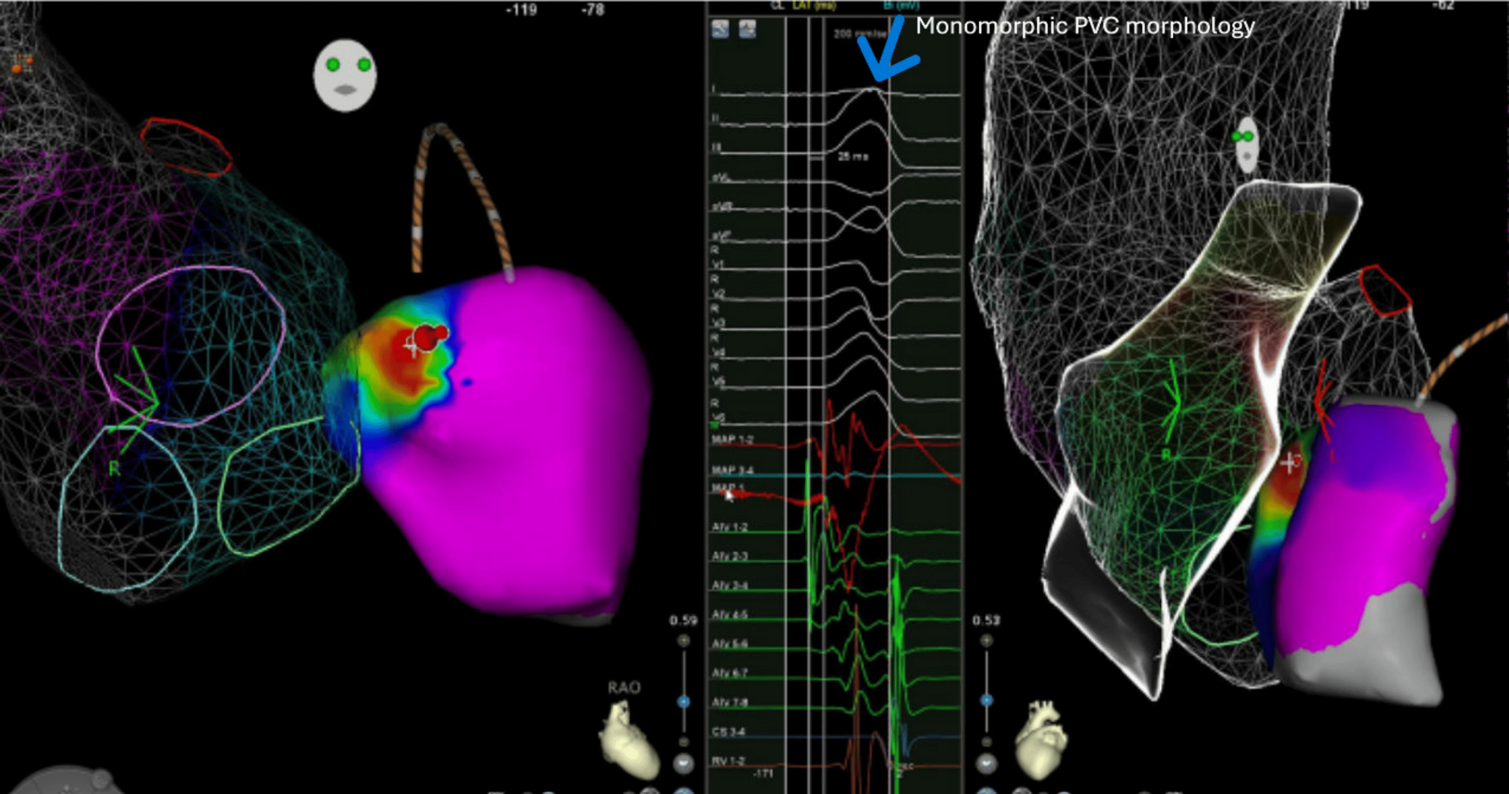
3D electroanatomical mapping of the left ventricle during catheter ablation for premature ventricular contractions (PVCs).

An electrophysiological study revealed monomorphic PVCs originating from the left ventricular summit (an anatomically small, triangular region at the most superior portion of the left ventricle, just beneath the left coronary artery bifurcation). This area was targeted for ablation, utilizing a power of 50 Watts and a temperature under 32°C for the total duration of 5.4 minutes (Figure 4).

Three months later, a repeat echocardiogram showed improvement in the LVEF to 40%-45% and repeat 14-day heart monitoring demonstrated a reduction in PVC burden to below 1%.

At three months post-PVC ablation, the patient was also found to be in new persistent atrial fibrillation with controlled ventricular rate, which was refractory to medication and symptomatic. Three months after that, he underwent cryoablation utilizing advanced mapping with successful pulmonary vein isolation. Following the procedure, his blood pressure improved, allowing for the addition of empagliflozin to his medical regimen. Repeat echocardiography one year post-PVC ablation showed further improvement in his LVEF to 50%-55%. Cardiogenetic testing was performed for a panel of arrhythmia and cardiomyopathy genetic mutations, revealing no disease-causing deoxyribonucleic acid (DNA) variants in the tested genes. Echocardiography two years post-PVC ablation demonstrated normalization of his LVEF to 55%-60%.

## Discussion

PVCs are most often a benign clinical finding that tends to be more bothersome than dangerous. However, when PVCs occur with a high frequency (i.e., >10% PVC burden), they have been identified as a potential cause of dilated cardiomyopathy and heart failure, and in certain circumstances, PVC ablation has been shown to be effective in reversing cardiomyopathy [1]. The question of how high the PVC frequency must be to develop a cardiomyopathy has been debated. One study identified a PVC burden of at least 24% as a potential threshold above which the risk of cardiomyopathy increases [2]. In a subsequent study, PVC burdens as low as 0.123%-17.7% were associated with a three-fold increased risk of cardiomyopathy [3]. In most cases, a PVC burden of at least 10%-15% is felt to be the threshold at which concern for PVC-induced cardiomyopathy increases. Other contributing factors include PVC QRS duration >153 ms and non-outflow tract PVC origin [4].

The mechanism by which PVCs induce cardiomyopathy is not fully understood. One proposed mechanism is ventricular dyssynchrony resulting in electromechanical dysfunction, which may explain why longer PVC QRS durations are associated with increased incidence of cardiomyopathy. Another proposed mechanism is that PVCs result in left ventricular (LV) remodeling. This may be supported by the finding of reduced LV strain by speckle tracking in patients with preserved LV systolic function and frequent PVCs [5]. Interestingly, one animal model demonstrated that a cardiomyopathy could be induced by inducing frequent PVCs using an implanted pacemaker in dogs [6]. Using that same dog model, PVC-induced cardiomyopathy was present in 11.1%, 44.4%, and 100% of animals with 25%, 33%, and 50% PVC burden, respectively [7].

Treatment of PVC-induced cardiomyopathy has focused largely on the efficacy of catheter ablation. The success rate for reversing cardiomyopathy and restoring LV systolic function ranges from 47% [8] to as high as 100% (the latter when the PVCs originate in the right ventricular outflow tract (RVOT)) [9]. Recovery of LVEF typically occurs within four months post ablation, although it can take up to 45 months in some cases [10]. In our case, the PVCs originated from the LV summit. This location is often associated with idiopathic PVCs and can be more challenging to ablate. Beta blockers and class III antiarrhythmics such as amiodarone have also been studied as potential treatments for PVC-induced cardiomyopathy. However, ablation is felt to be superior to medical therapy in both suppression of PVCs and LVEF recovery. In one study, LVEF was restored in 47% of patients in the ablation group compared to only 21% of patients in the antiarrhythmic therapy group [8].

In our case, the patient initially presented with a 6% PVC burden and preserved LVEF. One and a half years later, he developed a 27% PVC burden and was found to have dilated cardiomyopathy. Before confirming the diagnosis of PVC-induced cardiomyopathy, extensive testing ruled out other causes, including ischemic cardiomyopathy, sarcoidosis, and infiltrative diseases. One potential confounder is the patient’s chronic alcohol use. Most studies suggest that a daily intake of 80 grams of alcohol, approximately one liter of wine, eight standard beers, or half a pint of hard liquor, is necessary to cause alcoholic cardiomyopathy [11]. Our patient’s intake was well below this threshold. His subsequent abstinence may have facilitated both his reduction in PVC burden and LVEF recovery. Alternatively, he may have had a pre-existing myocardial substrate that made him particularly vulnerable to moderate alcohol consumption, predisposing him to cardiomyopathy as well as both ventricular and atrial arrhythmias. This raises the question: Did the PVCs cause the cardiomyopathy, or did the cardiomyopathy lead to frequent PVCs? A second confounding factor was the development of atrial fibrillation. The patient's LVEF had initial improvement following his PVC ablation to 40-45%. Subsequent to atrial fibrillation ablation, his LVEF further improved to 50-55%. It would be reasonable to assume that the atrial fibrillation ablation contributed to some of the improvement in systolic function beyond that afforded by his PVC ablation. We treated the patient with a combination of catheter ablation, alcohol avoidance, and guideline-directed medical therapy. His time course to recovery of systolic function post ablation was in keeping with reported averages. Patients with PVC-induced cardiomyopathy who recover LV function after ablation should undergo continued surveillance for recurrence. After three years of treatment, our patient remains free of PVCs with preserved LV function.

## Conclusions

PVC-induced cardiomyopathy remains an underrecognized but important cause of reversible heart failure. This case underscores how an increased PVC burden can contribute to the development of cardiomyopathy in a previously healthy individual and illustrates the potential for meaningful recovery of left ventricular function following successful catheter ablation. A comprehensive evaluation to exclude alternative causes of cardiomyopathy is essential, especially in patients with potential confounding factors such as alcohol use. Our patient’s outcome emphasizes the value of early detection, close monitoring, and timely intervention, including lifestyle modification, guideline-directed medical therapy, and catheter ablation, in preventing irreversible myocardial dysfunction. Greater awareness of PVC-induced cardiomyopathy may enable clinicians to identify high-risk patients sooner, mitigate disease progression, and improve long-term outcomes.
